# Detection of volatile organic compounds using mid-infrared silicon nitride waveguide sensors

**DOI:** 10.1038/s41598-022-09597-9

**Published:** 2022-04-02

**Authors:** Junchao Zhou, Diana Al Husseini, Junyan Li, Zhihai Lin, Svetlana Sukhishvili, Gerard L. Coté, Ricardo Gutierrez-Osuna, Pao Tai Lin

**Affiliations:** 1 Department of Electrical and Computer Engineering, Texas A&M University, College Station, TX 77843 USA; 2 Department of Materials Science and Engineering, Texas A&M University, College Station, TX 77843 USA; 3 Department of Biomedical Engineering, Texas A&M University, College Station, TX 77843 USA; 4 Department of Computer Science and Engineering, Texas A&M University, College Station, TX 77843 USA

**Keywords:** Imaging and sensing, Optical sensors

## Abstract

Mid-infrared (mid-IR) sensors consisting of silicon nitride (SiN) waveguides were designed and tested to detect volatile organic compounds (VOCs). SiN thin films, prepared by low-pressure chemical vapor deposition (LPCVD), have a broad mid-IR transparent region and a lower refractive index (n_SiN_ = 2.0) than conventional materials such as Si (n_Si_ = 3.4), which leads to a stronger evanescent wave and therefore higher sensitivity, as confirmed by a finite-difference eigenmode (FDE) calculation. Further, in-situ monitoring of three VOCs (acetone, ethanol, and isoprene) was experimentally demonstrated through characteristic absorption measurements at wavelengths λ = 3.0–3.6 μm. The SiN waveguide showed a five-fold sensitivity improvement over the Si waveguide due to its stronger evanescent field. To our knowledge, this is the first time SiN waveguides are used to perform on-chip mid-IR spectral measurements for VOC detection. Thus, the developed waveguide sensor has the potential to be used as a compact device module capable of monitoring multiple gaseous analytes for health, agricultural and environmental applications.

## Introduction

Detection of VOC is critical for a variety of applications, ranging from health diagnostics to environmental and industrial monitoring^1–5^. Two general approaches are often used for VOC analysis, one based on analytical techniques such as gas chromatography mass spectrometry (GC–MS)^6,7^, and another involving solid-state sensors, based on metal-oxide semiconductors (MOS)^8–10^, electrochemical (EC)^11^ or photoionization detection (PID)^12^. GC–MS can provide accurate gas analysis, but the systems are bulky and thus unsuitable for point-of-use (POU) and real-time applications. In turn, solid-state chemical sensors can have high sensitivity and portability, but have low selectivity to discriminate among VOCs. As an alternative to conventional solid-state sensors, mid-IR sensing can provide high selectivity by measuring absorption of the characteristic and finger-print vibrational features of VOCs. However, mid-IR absorption spectroscopy requires bench-top optical equipment such as Fourier-transform infrared spectroscopy (FTIR), which is unpractical for POU applications^13,14^. To address this issue, miniaturized photonic circuits consisting of optical waveguides and other chip-scale photonic components have been explored^15–17^. However, prior approaches have used waveguide materials that have high refractive indices, such as Si and Ge^18,19^, which lead to a weak evanescent wave^20,21^ and therefore poor sensitivity. Chalcogenide materials have also been used^22^, and they provide stronger evanescent fields, but they are prone to degradation upon exposure to moisture, thus requiring storage under dry N_2_ or high vacuum condition.

To address this issue, the present study examines the use of SiN as the waveguide material. SiN has a low refractive index (**n**_**SiN**_ = 1.94) compared to Si (**n**_**Si**_ = 3.4), which leads to a strong evanescent wave and therefore higher sensitivity, and also possesses exceptional chemical stability and can be repeatedly used in ambient humidity conditions^23,24^. Further, SiN has a broad infrared transparency window, low optical loss, and high compatibility with complementary metal-oxide-semiconductor (CMOS) processes^25,26^. These properties make SiN an ideal material for waveguide sensors to enable repeatable, reproducible VOCs detection over long-term sensing operation. To our knowledge, however, prior work on SiN as a waveguide material has been primarily theoretical or has focused on wavelength sensing in the visible range, rather than the far more informative mid-IR range^27–29^.

To demonstrate the potential of SiN waveguide sensing for mid-IR detection of gaseous analytes, we focused on three VOCs (acetone, ethanol, and isoprene) that are potential biomarkers for breath analysis. For instance, exhaled acetone has been studied to monitor diabetic ketoacidosis^30,31^, ethanol for alcohol abstinence^32^, and isoprene for lung cancer and high blood cholesterol^33,34^. Further, these VOCs have strong mid-IR absorption signatures at wavelengths between 3.0–3.6 μm, which are detectable using the proposed SiN photonic circuits. Being compatible with CMOS fabrication processes, the proposed SiN mid-IR waveguide sensor offers the potential for a compact device platform to realize real-time gas analysis.

## Experimental methods

### Fabrication of SiN waveguide sensor and gas chamber

**Figure 1:**
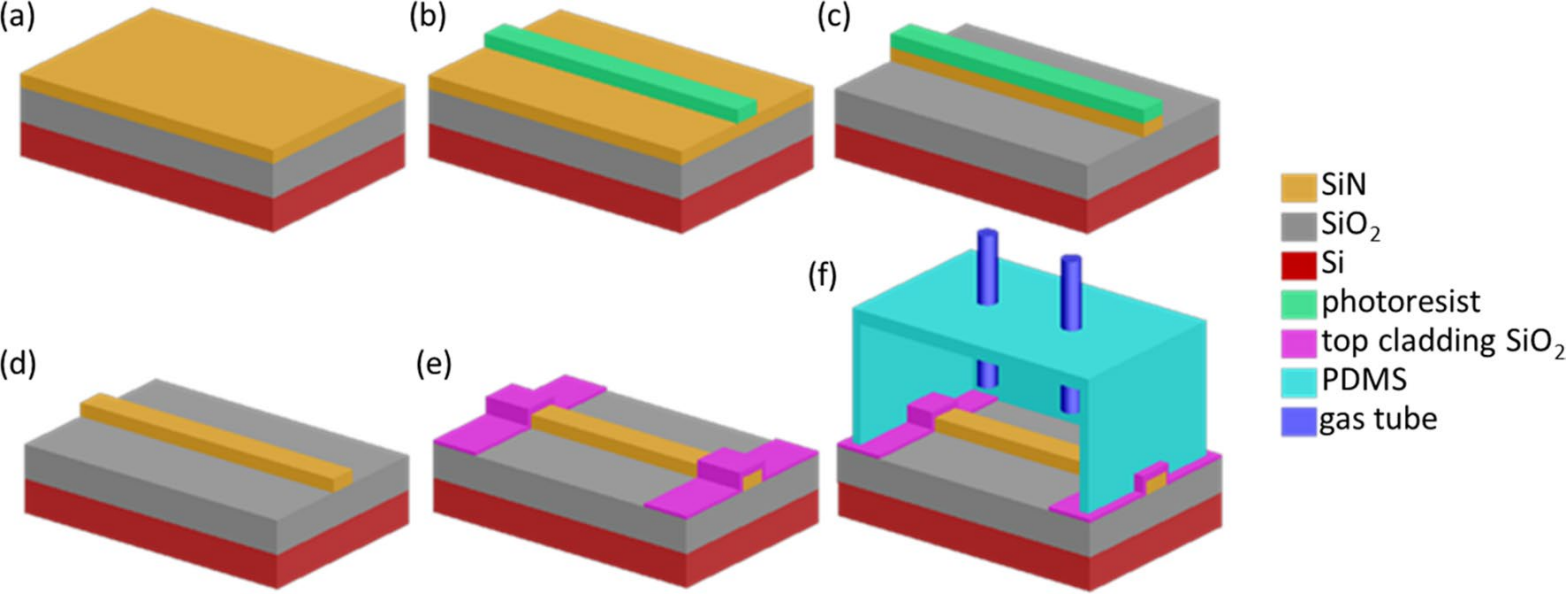
Fabrication process of the SiN waveguide and its assembly with a PDMS chamber. (**a**) Deposition of SiN thin film by LPCVD. (**b**) Creation of waveguide patterns by photolithography. (**c**) Transfer of the waveguide pattern to the SiN layer by RIE. (**d**) Removal of photoresist. (**e**) Deposition of the top SiO_2_ cladding layer. (**f**) Bonding of the PDMS chamber to the waveguide sensor.

Figure 1a–f illustrates the fabrication of mid-IR SiN waveguides, along with a polydimethylsiloxane (PDMS) chamber for VOC detection. In step (a), a low-stress SiN thin film with thickness T = 1 μm was deposited on a Si wafer by LPCVD. The grown Si-rich SiN has a low tensile stress of 45 MPa. A 3 μm thick thermo-oxide acts as the low-refractive-index under-cladding layer between the SiN and the Si. In step (b), the pattern of waveguides was generated on top of the SiN thin film via photolithography (Karl Suss MA-6 mask aligner). The waveguide pattern was then transferred into the SiN thin film layer in step (c) using reactive ion etching (RIE). After removing the photoresist in step (d), a 3 μm thick SiO_2_ layer was deposited on both sides of the SiN waveguide by plasma-enhanced chemical vapor deposition (PECVD). The center of the waveguide was left open for VOC detection, as shown in step (e). The length of the waveguide sensing region, where the mid-IR evanescent wave interacted with the VOCs, is 8 mm long. A PDMS (Dow Corning Sylgard 184) chamber was prepared by dropping the PDMS precursor into a customized mold. In step (f), the molded PDMS was bonded to the SiN waveguide device to form a sealed gas chamber. The 8 mm long sensing region is an ideal length to integrate with the PDMS chamber providing high gas sealing. The top SiO_2_ layer patterned in step (e) eliminates direct contact between the PDMS chamber and the SiN waveguides to prevent absorption by PDMS from interfering with the sensing measurement.

### Setup for SiN waveguide characterization and VOC absorption measurement

**Figure 2:**
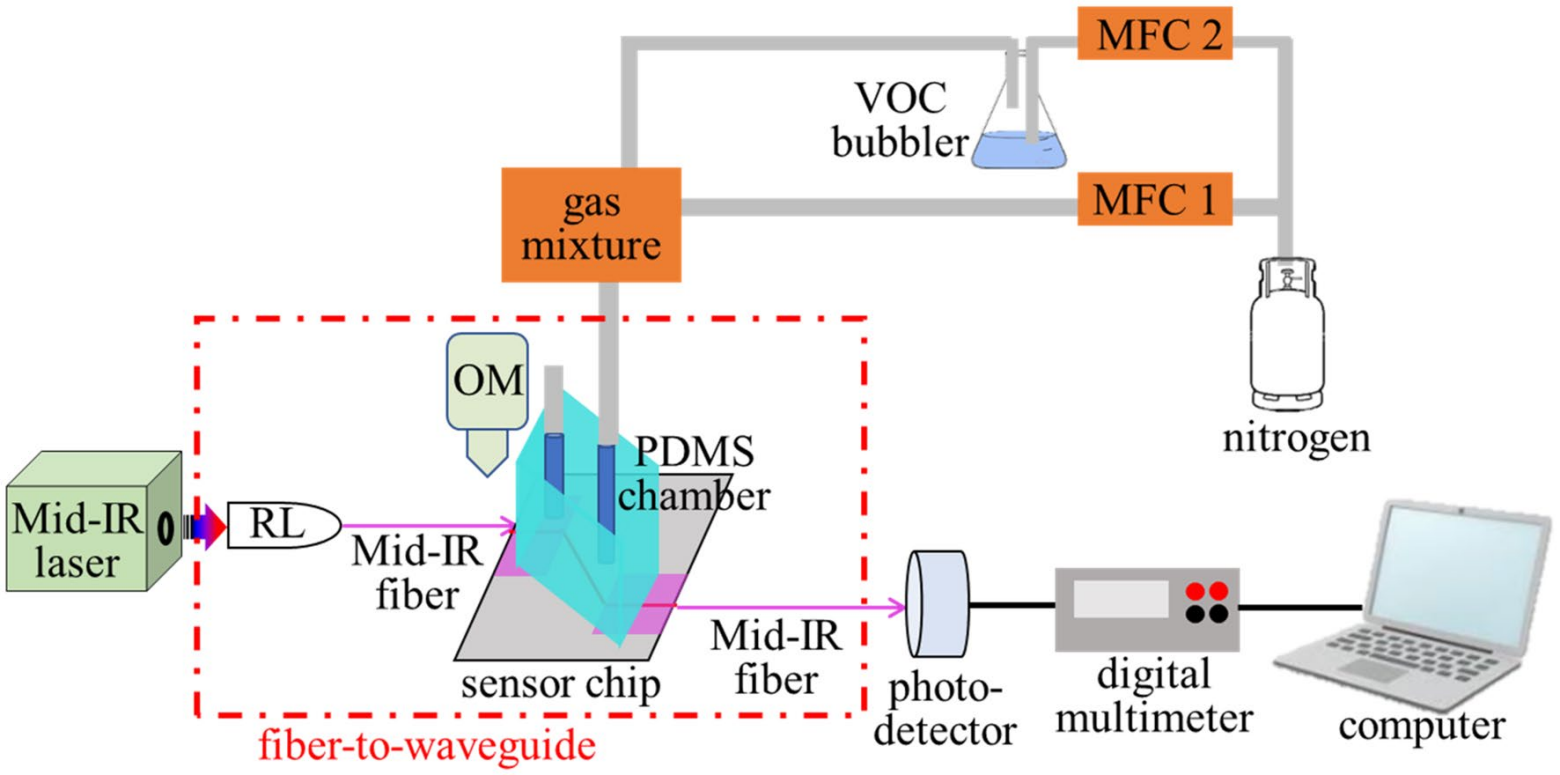
Experimental setup of the VOC detection measurement. Mid-IR probe light from the laser was coupled into the waveguide using a butt-coupling method. The guided light was collected by another fiber after the waveguide end-facet. VOC vapor was carried by N_2_. The flow rate the VOC concentration were controlled by the MFCs.

Figure 2 shows a schematic of the experimental set-up for waveguide characterization and VOC detection via a fiber-waveguide-fiber configuration. A tunable mid-IR laser (M Squared Firefly) with 150 kHz pulse repetition rate, 10 ns pulse duration, and 150 mW average power, was used as the light source. The wavelength tuning range of the laser was from 2.5 to 3.7 µm. The laser light was coupled into a single mode ZrF_4_ fiber (Thorlabs) through a reflective lens collimator (RL) and then butt-coupled to the SiN waveguide. The fine position adjustment between the fiber and the waveguide was monitored with an optical microscope. The coupling loss was 5.2 dB and the waveguide loss was 2.1 dB/cm at 3.5 μm wavelength.

Three VOCs (acetone, ethanol, and isoprene) were used as the analytes. VOC vapors were prepared by flowing nitrogen gas through bottles with VOCs in liquid form. The flow rates of the VOC vapors and the nitrogen carrier gas were regulated by mass flow controllers (MFCs). The gas phase analyte was delivered into the sealed PDMS chamber with the waveguide sensor inside. The gas pressure into the PDMS chamber was monitored by the mass flow controllers and pressure regulators. The temperature of the system was maintained at room temperature 20 °C. The intensity of the waveguide light was attenuated according to the mid-IR absorption spectra of the VOCs. Light propagating through the waveguide was collected by a multimode ZrF_4_ fiber with a 400 μm core diameter (Thorlabs). The fiber was connected to a photodetector (Thorlabs PDA20H), and the light intensity was read by a digital multimeter and recorded in a computer.

## Results and discussion

### Characterization of SiN waveguides and VOCs absorption measurement

**Figure 3:**
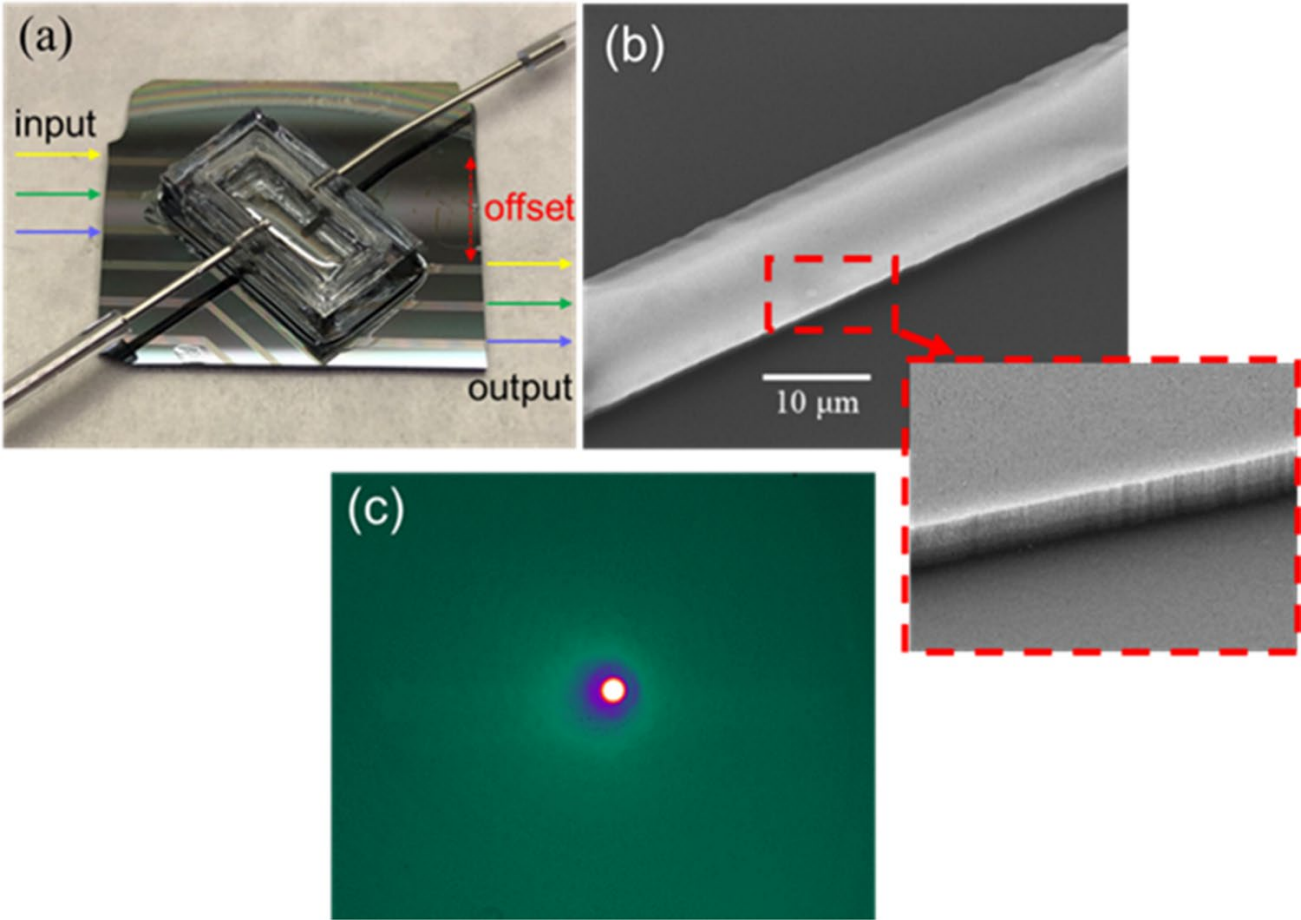
(**a**) Sensor device with SiN waveguides and PDMS chamber. Input and output waveguides were offset by 5 mm. (**b**) SEM top image of a SiN waveguide. The zoom-in tilted image shows its side wall. (**c**) The mode image from a single SiN waveguide.

Figure 3a shows the fabricated SiN waveguide sensor with the attached PDMS gas chamber. The input and output waveguides were placed out of line with an offset of 5 mm to minimize contributions from background light that was not coupled into the waveguide. Figure 3b is an SEM image of the SiN waveguide and its die wall. The waveguide width is 10 μm. The reason of using the 10 μm wide waveguide is to improve the coupling efficiency between the 9 μm core-diameter fiber and the SiN waveguide. Both the waveguide top surface and side wall are smooth, as depicted in Fig. 3b. Figure 3c shows the waveguide mode of the SiN waveguide captured at λ = 3.3 µm by a liquid-nitrogen cooled mid-IR camera (IRC 800 series, IRcameras). A sharp spot corresponding to a single fundamental waveguide mode was observed, and no scattering was found in the entire field of view. This indicates that the input light was well confined within the SiN waveguide, and that the background light in the substrate layer was minimized by the off-set design. Specifically, the width of the fabricated waveguide is 10 μm, which is close to the single mode mid-IR fiber with a 9 μm diameter. Therefore, the waveguide mode excitation efficiency for the fundamental mode is larger than the higher-order modes because the fundamental mode has a better spatial overlap with the fiber’s single-mode. As a result, the experimentally observed waveguide mode showed a fundamental mode dominant profile. Our LPCVD Si-rich SiN also has low optical loss of 0.2 dB/cm and low tensile stress of 45 MPa for a film thickness of 2 μm.

### Finite-difference eigenmode (FDE) analysis of waveguide modes and mid-IR sensitivity

**Figure 4:**
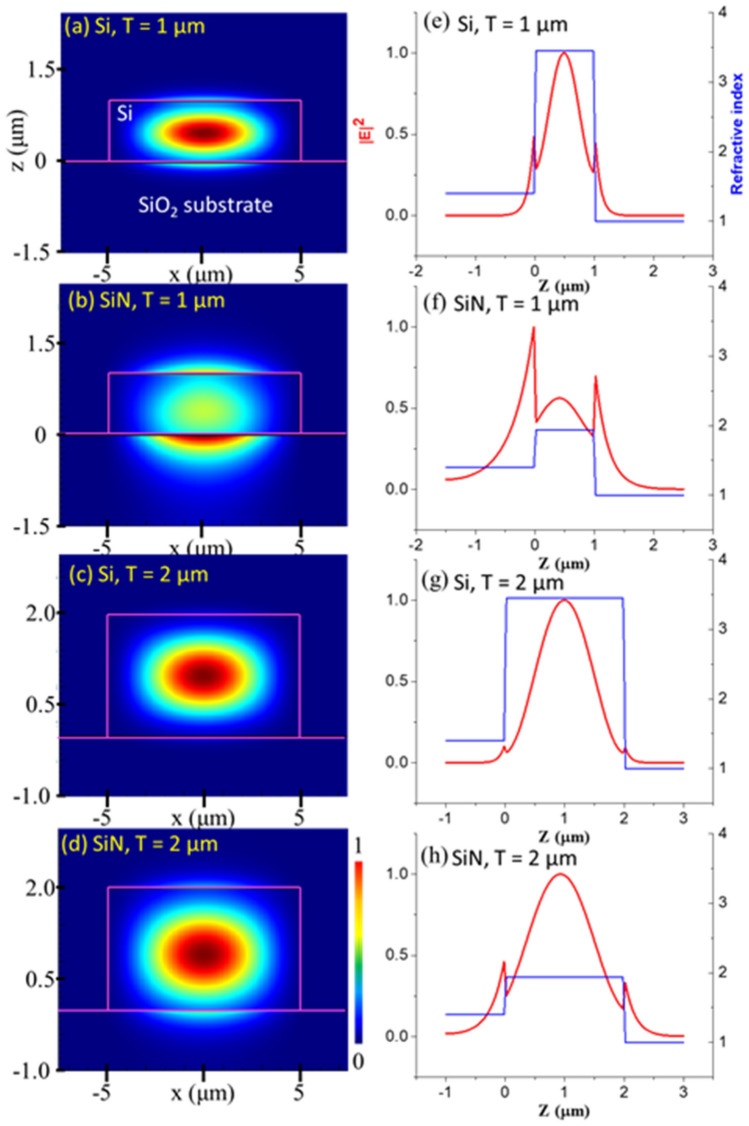
(**a**–**d**) Simulated 2-D waveguide modes and (**e**–**h**) 1-D intensity profiles along the z-axis for thicknesses T = 1 and 2 µm for the Si and SiN waveguides, respectively. TM polarization was selected for the waveguide modes.

Mode profiles of the SiN and Si waveguides and their sensitivities were calculated using FDE, which allowed us to evaluate the sensitivity of mid-IR waveguides made of different materials and structures. Figure 4a–d shows the 2-D waveguide modes for various waveguide structures at λ = 3.3 μm, and Fig. 4e–h displays the corresponding 1-D light intensity and refractive indexes distributions along the z-axis at x = 0 μm. The waveguides had the same width of 10 μm. Two different waveguide thicknesses of T = 1 μm and 2 μm were considered for the transverse magnetic (TM) polarization. Waveguide sensing performance was evaluated by comparing the evanescent field intensity distribution for the two waveguides. The T = 1 μm Si waveguide showed a weak evanescent field extending to the outer medium (z > 1 μm), while the T = 1 μm SiN waveguides showed a strong evanescent field outside of the waveguide. The intensity profile remained the same in the *x* direction. As the thickness of the waveguide increased to T = 2 μm, the intensity of the evanescent field significantly decreased for both the SiN and Si waveguides, as illustrated in Fig. 4c,d, respectively. However, the SiN waveguide still showed a stronger field than the Si waveguide.

**Figure 5:**
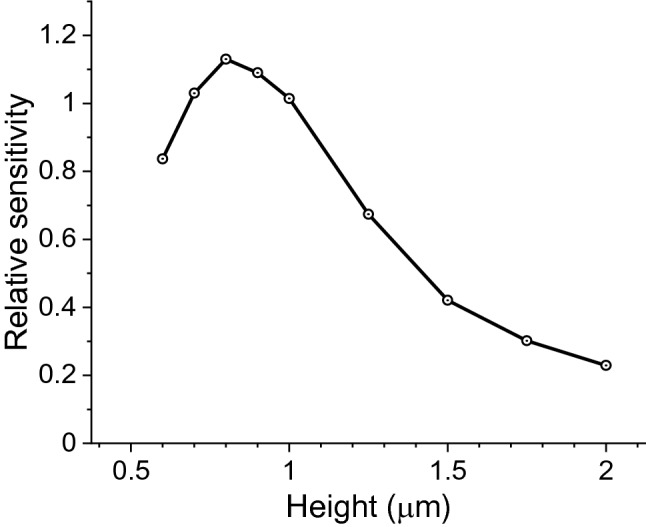
Sensitivity vs waveguide thickness T at **n** = 2, which corresponds to the SiN material.

**Figure 6:**
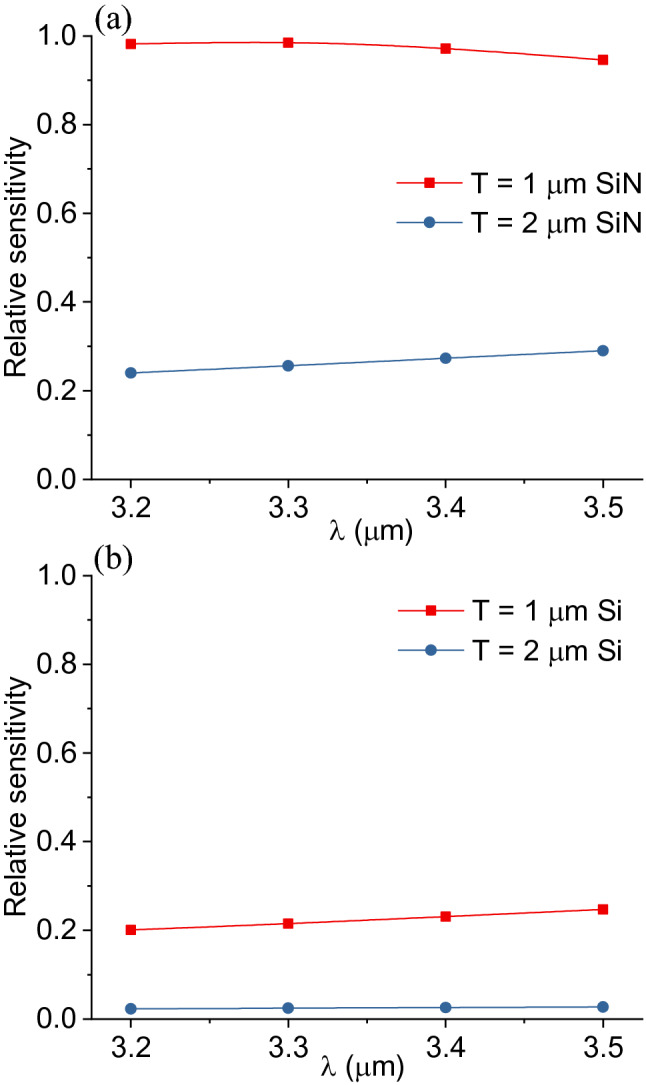
Sensitivity vs wavelength (λ) for (**a**) SiN and (**b**) Si waveguides at thicknesses T = 1 and 2 μm, respectively.

The FDE method was also used to analyze the sensitivity of the mid-IR waveguides. The sensitivity of the waveguide is defined as the attenuation of the waveguide intensity, which is equivalent to the waveguide propagation loss per unit length, upon exposure to an external medium. Here, the external medium is the VOC analyte and it has a homogeneous refractive index profile. The real part of the refractive index of the external medium is 1 and the imaginary part is determined by the absorption coefficient of the VOC. The relative sensitivity was then calculated by comparing its value with the sensitivity at T = 1.0 μm. The wavelength in the simulation is λ = 3.375 μm. As shown in Fig. 5, the sensitivity was improved by 5.1 times as the thickness T decreased from 2 to 0.8 μm. This was because the evanescent field extended into the VOC analyte increased at the thinner waveguide thickness. The sensitivity decreased at T < 0.8 μm because such a thin waveguide no longer supported the waveguide mode. The sensitivity vs. wavelength was also investigated and illustrated in Fig. 6. The change of sensitivity was almost negligible for both waveguide materials across the 3.2–3.5 μm wavelength range, indicating the waveguide sensors have uniform performance over a broad spectrum, which is critical for mid-IR VOCs analysis.

### Real-time VOC detection and absorption spectrum characterization

**Figure 7:**
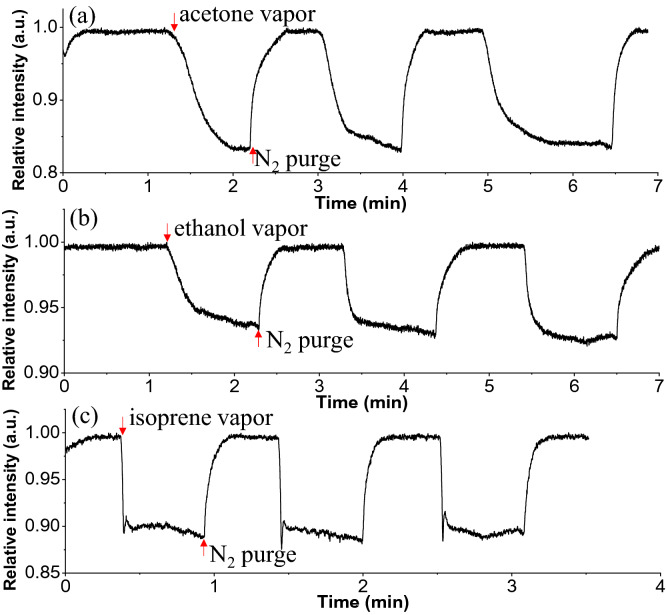
Real-time mid-IR monitoring of pulsed VOCs using SiN waveguides of width = 10 µm and thickness = 1 µm: (**a**) acetone at λ = 3.375 µm, (**b**) ethanol at λ = 3.375 µm, and (**c**) isoprene at λ = 3.400 µm.

Figure 7 shows real-time VOC detection using the mid-IR SiN waveguide sensor illustrated in Fig. 2. The SiN waveguide had a width of 10 μm and a thickness of 1 μm. The waveguide thickness, T, was optimized and fixed to 1 μm for both the Si and SiN waveguides due to the limitation in coupling from the large 9 μm core fiber. A waveguide thinner than 1 μm will decrease the fiber-to-waveguide butt-coupling efficiency, since presently there is no conventional mid-IR lensed fiber available. Although other coupling approaches such as on-chip light sources and off-axis parabolic mirror are under development, this butt-coupled fiber optic approach is what is currently commercially available with high coupling efficiency. During the measurement, pure nitrogen was first injected into the PDMS chamber, and the waveguide intensity obtained was defined as the baseline. Once the waveguide intensity stabilized, VOC vapor was injected into the PDMS chamber until the waveguide intensity stabilized. Each measurement was repeated for 3–4 cycles of VOC injection and nitrogen purging to ensure the results were consistent and repeatable. The transient response during the acetone, ethanol, and isoprene measurements are plotted in Fig. 7a–c, respectively, and showed a deviation over time within 1% for each of the gases.

The vibrational bands caused by the C–H stretching vibrations for acetone are 3.305 μm with A1 symmetry and 3.168 μm with F2 symmetry. For ethanol, there are symmetric stretching vibration at 3.412 μm and symmetric stretching vibration at 3.364 μm. For acetone and ethanol vapor, the waveguide light intensity decreased over time when their vapor flowed into the chamber and then reached an equilibrium state after 10–20 s, indicating that the VOC molecules steadily attached to the waveguide surface. Once the nitrogen was reinjected into the chamber, the waveguide intensity increased and recovered to its initial intensity level within 20 s. For isoprene, the waveguide light intensity dropped nearly instantaneously once the isoprene vapor was injected due to its high volatility.

**Figure 8:**
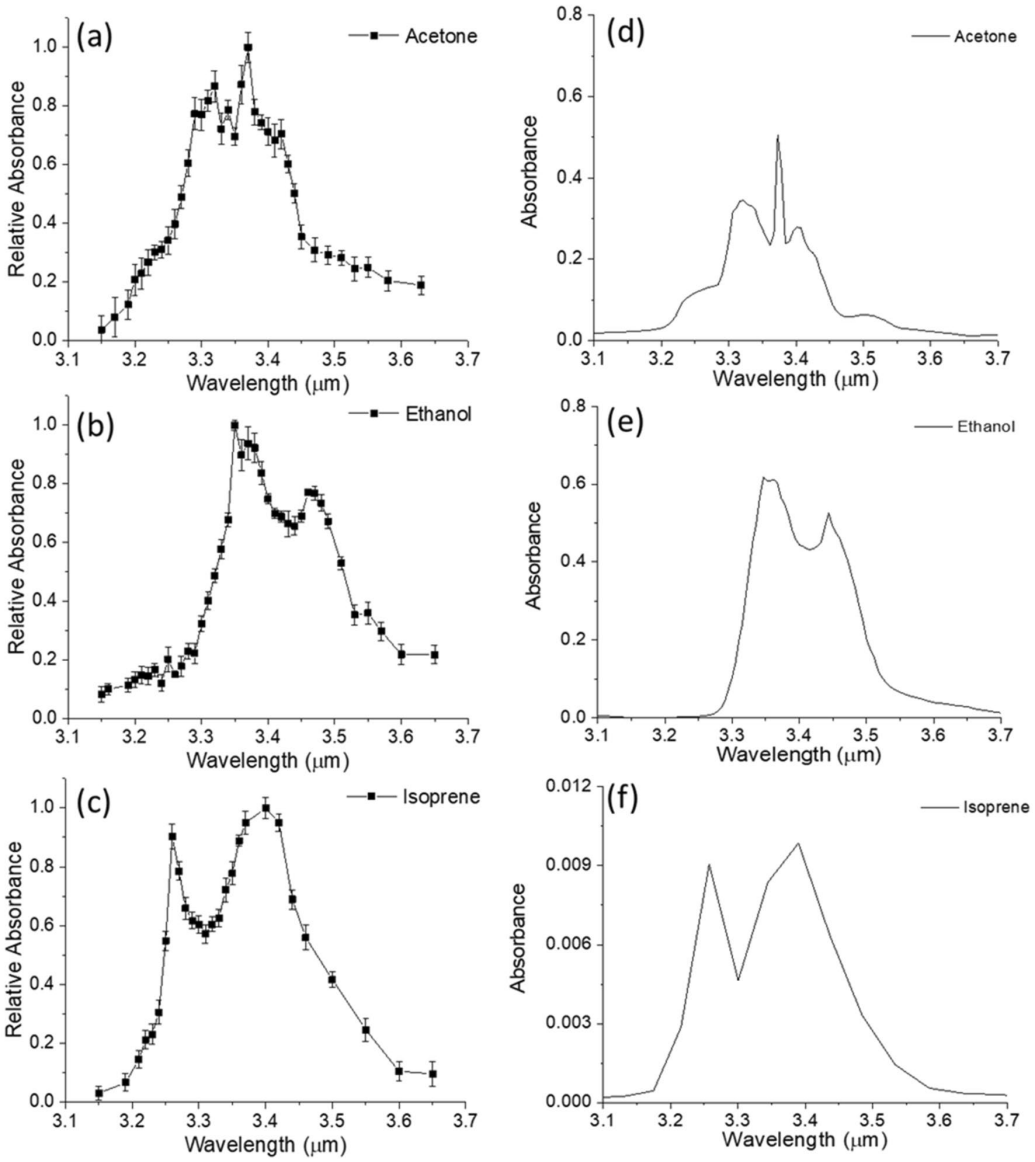
Mid-IR absorption spectra of acetone, ethanol, and isoprene. (**a**–**c**) Results from SiN waveguide measurement. (**d**–**f**) Spectra from the NIST WebBook database^35^.

**Figure 9:**
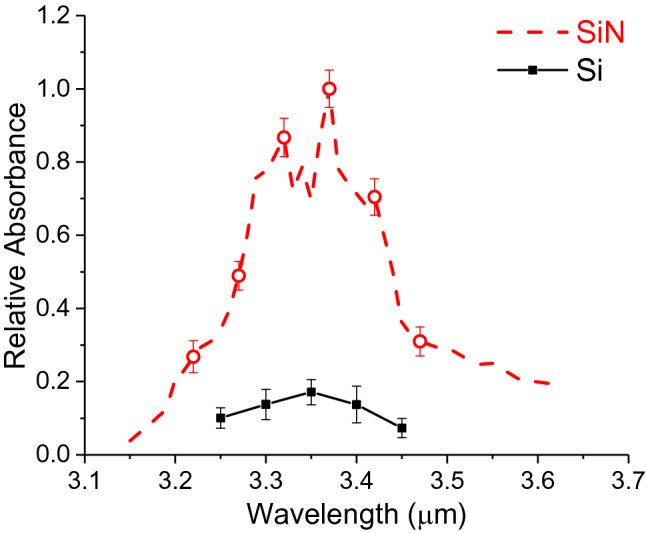
Measured results of acetone using SiN waveguides compared with Si waveguides. The waveguide width = 10 μm and thickness T = 1 μm.

These results indicate that the SiN waveguide can detect different VOCs and measure their transient response. Unlike chalcogenide waveguides, SiN has high chemical stability and resistance to moisture, so the SiN waveguide sensor does not demonstrate signal degradation and can be used for long-term repeatable VOCs monitoring. The absorption spectrum of VOCs can be calculated using the real-time measurement according to the Beer's law equation A = − log(I/I_0_), where A is the absorbance, and I and I_0_ are the waveguide intensities with and without the VOC, respectively. The spectra of acetone, ethanol and isoprene measured by the SiN waveguide are plotted in Fig. 8a–c, where the absorbance in y-axis is normalized to its maximum. Note that the spectra are not meant to show the ability to separate these gas molecules spectrally but rather to show that the characteristic absorption bands caused by the C–H stretching vibrations between λ = 3.1 and 3.6 μm could be observed. The spectral features were consistent with FTIR spectra (NIST WebBook) shown in Fig. 8d–f, though the resolution of our tunable laser was not as high. For instance, the major absorption peak at λ = 3.35 μm was depicted for acetone and at 3.4 for μm ethanol for our system. In addition, the two broader isoprene absorption peaks at λ = 3.25 and 3.4 μm were observed in both systems. Figure 9 shows the relative absorbance of acetone using a Si waveguide, compared to the results obtained using the SiN waveguides. Both Si and SiN waveguides were 10 μm wide and 1 μm thick in these experiments. The results of Fig. 9 experimentally confirm the primary result anticipated from the FDE modeling, showing that attenuation (and therefore sensitivity) in the SiN waveguide is five times stronger than that of the Si waveguide.

**Figure 10:**
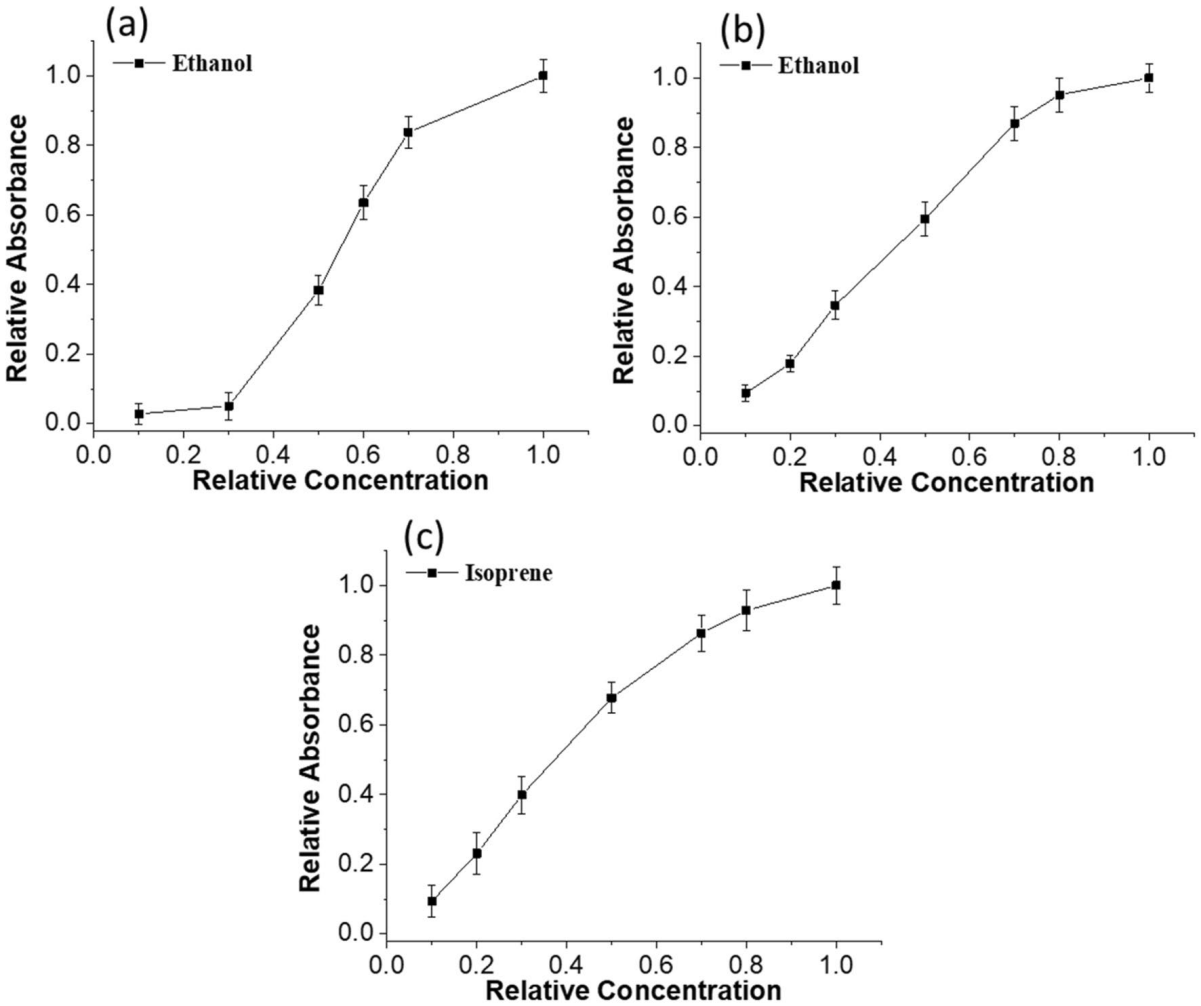
Relative absorbance vs relative VOC concentration for (**a**) acetone at λ = 3.375 µm, (**b**) ethanol at λ = 3.375 µm, and (**c**) isoprene at λ = 3.400 µm.

Figure 10 shows the relative absorbance with relative concentration of the individual VOCs. Gas concentration was controlled by adjusting the flow rates of MFC 1 and MFC 2; see Fig. 2. The x-axis in Fig. 10 is the ratio of flow rates: (MFC2)/(MFC1 + MFC2). We observe a monotonic increase in relative absorbance with VOC concentration, which is fairly linear below a ratio of 0.7 and then follows a nonlinear increase at higher ratios. The absorption curve can be explained by a superposition of an adsorption isotherm and a linear adsorption increase caused by the non-adhesive analytes within the waveguide’s evanescent field. Applying the Antoine equation, the concentration of the VOC acetone vapor is 10 mmol/L, equivalent to 24% at 1 atm^36^. Further, according to the Lambert–Beer’s Law and using the sensitivity as defined in Ref.^38^, the waveguide output intensity *I* can be derived from Eq. (1).

1 $$ I = I_{0} e^{ - A} = I_{0} e^{ - \varepsilon \eta cl} $$

Here, *I*_0_ is the waveguide output intensity without VOCs, *A* is the VOC absorbance, $\varepsilon$ is the specific VOC absorption, $\eta$ is the fraction of the evanescent power outside of the waveguide core, *c* is the VOC concentration, and *l* is the sensing length. The ultimate theoretical sensitivity of the waveguide is then defined in Eq. (2).

2 $$ S = \frac{dI}{{dc}} = - I_{0} \varepsilon \eta le^{ - \varepsilon \eta cl} $$

From the real-time measurement in Fig. 7, $A_{max} = - \log \left( {\frac{I}{{I_{0} }}} \right) = - \log \left( {\frac{0.85}{1}} \right) = 0.071$, which is the maximum absorbance upon exposure to the saturated acetone vapor. The noise equivalent power (NEP) of the mid-IR photodetector is $1.5 \times 10^{ - 10}\, {\text{W}}/\sqrt {{\text{Hz}}}$, and the responsivity is $3 \times 10^{3}\, {\text{V}}/{\text{W}}$. The integration time is 35 μs, resulting in a bandwidth of $\Delta f = \frac{1}{{2t_{c} }} =$ 14 kHz. Considering the above parameters, the noise level *I*_noise_ is ± 0.05 mV. Because the waveguide output intensity is higher than 100 mV, the ratio of the noise compared to the waveguide output intensity is 0.1%. Therefore, the signal fluctuation is mainly caused by the circuit noise from the photodetector. In addition, the signal to noise ratio should be at least 3 for a valid absorption measurement. This corresponds to a detectable signal change of $\Delta I = \pm$ 0.3% *I*_0_ or a minimum detectable absorbance $A_{min} = - \log \left( {\frac{{I_{0} - \Delta I}}{{I_{0} }}} \right) = 0.003$. Hence, the LOD of acetone is 1/20 × the saturated acetone vapor, equivalent to 1.2%. Considering Eq. (1), we found $\alpha = \varepsilon \eta l = \frac{A}{c} = 0.3$. Therefore, the sensitivity at the lowest concentration of 1.2% is 30 mV per unit concentration change. Using the same approach, the LOD of the concentration were 1.3% for ethanol and 2.5% for isoprene. The sensitivity of our mid-IR waveguide is better than the refractive-index-based sensing using a visible waveguide because of the strong characteristic mid-IR C–H vibrational absorption^37^.

In the future, our waveguide sensitivity can be further increased with the use of slot waveguides and nanoparticles^39,40^. Specifically, our previous work has shown a 10–15 times enhancement of the sensitivity by coating nanoparticles on the device surface^41,42^. Further, compared to a ridge waveguide, a slot-waveguide can improve the sensitivity approaching 50 times. We expect that these advancements can enhance sensitivity by 500 ×, bringing it closer to that of MOS sensors, which achieve sub ppm levels, while still providing the advantage of better specificity. Thus, the potential for enhancement of sensitivity of waveguide-based sensing, taken together with intrinsically high, spectroscopic selectivity of mid-IR detection^43^ make this approach promising for future sensing applications of multiple gaseous analytes.

## Conclusions

Mid-IR Si and SiN waveguide sensors were evaluated theoretically and experimentally as robust, chemically stable platforms for on-chip VOC detection. Utilizing the C–H stretching vibrational mid-IR region between 3.1 to 3.5 μm, acetone, ethanol, and isoprene vapors were detected in real time using both waveguides. We demonstrated that due to their low refractive index, SiN waveguides had five times higher sensitivity than Si waveguides, and unlike Si waveguides were able to resolve the mid-IR absorption spectra of VOCs in the entire C–H strength region. Because of its CMOS compatibility, the proposed waveguide sensor can be integrated with wireless electronics and potentially provide a compact module for sensing of gaseous analytes for health, agricultural and environmental applications.
